# Supplementary material for: Climate Change Increases the Expansion Risk of Helicoverpa zea in China According to Potential Geographical Distribution Estimation
Source: Insects. 2022 Jan 11;13(1):79. doi: 10.3390/insects13010079 (PMC8781938; doi:10.3390/insects13010079)
Supplement: Supplementary file 1 [file insects-13-00079-s001.zip › insects-1482223-supplementary.pdf]

Supplementary Materials

Table S1 AUC values of the MaxEnt model results for different years

period	current	2030s SSP1-2.6	2050s SSP1-2.6	2030s SSP2-4.5	2050s SSP2-4.5	2030s SSP5-8.5	2050s SSP5-8.5
AUC	0.892	0.895	0.893	0.896	0.919	0.894	0.894
Stddev	0.002	0.001	0.003	0.003	0.001	0.004	0.003

Table S2 Suitable habitat areas for *Helicoverpa zea* under current conditions and different climate change scenarios (10^4 km^2)

Period	Poorly suitable habitat	moderately suitable habitat	highly suitable habitat	total suitable habitat
current	321.44	93.45	3.26	418.15
2030s, SSP1-2.6	301.3	88.01	5.97	395.28
2050s, SSP1-2.6	346.59	91.76	25.39	463.74
2030s, SSP2-4.5	303.48	89.38	4.58	397.44
2050s, SSP2-4.5	298.76	63.5	18.19	380.45
2030s, SSP5-8.5	295.1	88.4	5.43	388.93
2050s, SSP5-8.5	318.34	89.91	16.88	425.13

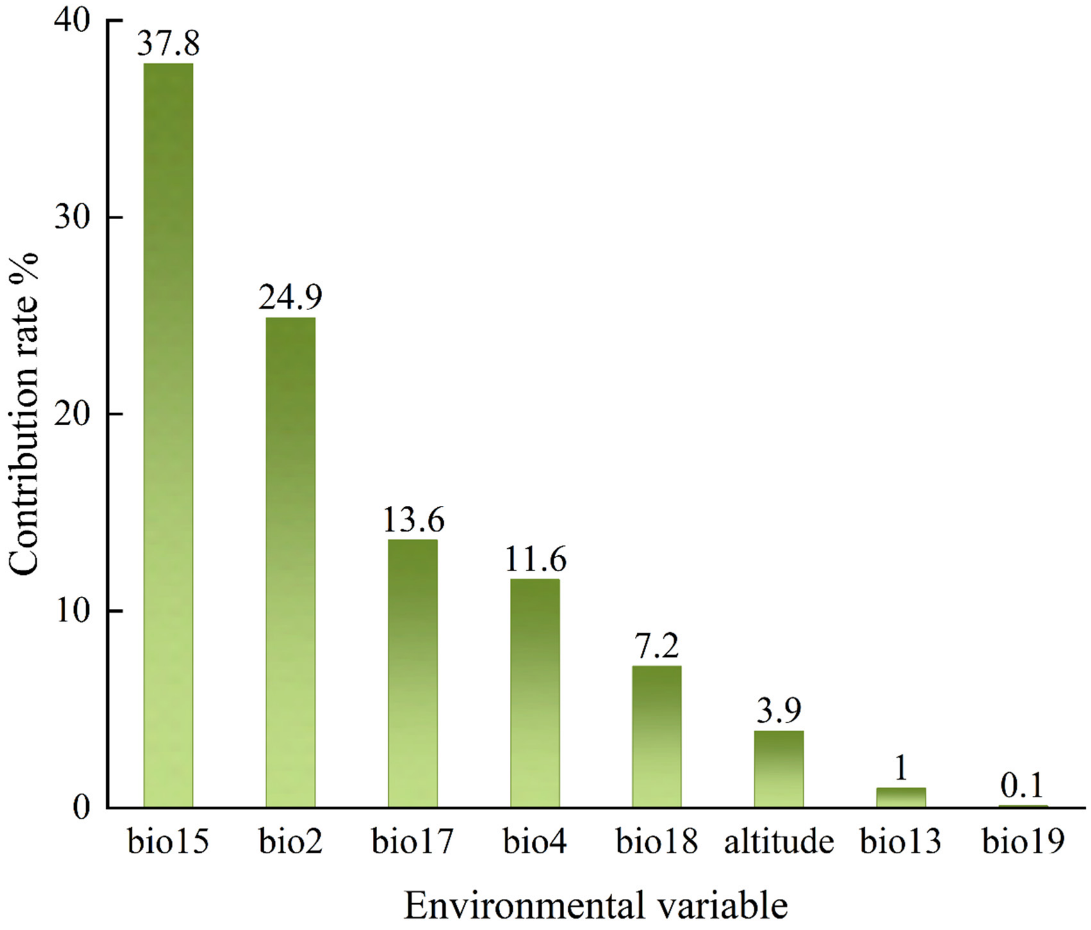

Figure S1. Influence of percentage contribution provided by 8 environmental variables based on the MaxEnt model.

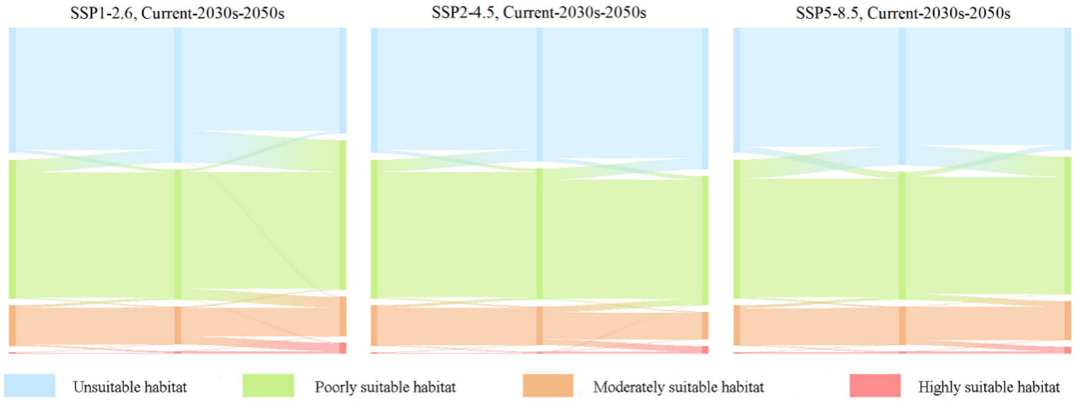

Figure S2. Area conversion between potential suitable habitats of *Helicoverpa zea* under current climate and project climate changes.
